# Cases Referred from the Turkish National Screening Program: Frequency of Congenital Hypothyroidism and Etiological Distribution

**DOI:** 10.4274/jcrpe.galenos.2019.2018.0255

**Published:** 2019-09-03

**Authors:** Zeynep Donbaloğlu, Şenay Savaş-Erdeve, Semra Çetinkaya, Zehra Aycan

**Affiliations:** 1 Dr. Sami Ulus Obstetrics and Gynecology, Children’s Health and Disease Training and Research Hospital, Clinic of Pediatrics, Ankara, Turkey; 2 Dr. Sami Ulus Obstetrics and Gynecology, Children’s Health and Disease Training and Research Hospital, Clinic of Pediatric Endocrinology, Ankara, Turkey

**Keywords:** Congenital hypothyroidism, neonatal screening program, newborn, thyroid hormones

## Abstract

**Objective:**

The aim of this study was to evaluate cases referred from the congenital hypothyroidism (CH) newborn screening program.

**Methods:**

Infants referred to Pediatric Endocrinology between 30.09.2015 - 01.04.2018 because of suspected CH identified by National Neonatal Screening Program were prospectively evaluated.

**Results:**

Of the 109 newborns referred to our clinic, 60 (55%) were diagnosed with elevated neonatal thyroid stimulating hormone (TSH). The diagnosis of elevated neonatal TSH was made in 52 (47.7%) and eight (7.3%) infants at initial evaluation and after follow up, respectively of all referrals with 86.7% (52/60) diagnosed at initial visit. The median first and second heel prick times were 1.8 (0-7) and 8.72 (4-30) days. The median age at starting treatment of the infants diagnosed as a result of initial evaluation was 22.13 (7-53) days. Clinical findings associated with CH were present in 19 (36%) of patients. Etiology in patients diagnosed with elevated neonatal TSH on admission was: agenesis in one (2.08%); ectopia in one (2.08%); hypoplasia in 14 (29.16%); normal gland *in situ* 16 (33.3%); and hyperplasia in 16 (33.3%). The median time to normalization of TSH and free thyroxine concentrations after treatment initiation was 11.02 (4-30) and 9.03 (3-30) days, respectively.

**Conclusion:**

The rate of diagnosis in the first month was found to be 87%. The etiological incidence of both dysgenesis and dyshormonogenesis was equal at 33.3%. The majority of cases with normal thyroid gland will be diagnosed with transient hypothyroidism but some of them may be diagnosed with thyroid dyshormonogenesis so the rate of dyshormonogenesis will increase later after final diagnosis.

**What is already known on this topic?**

Congenital hypothyroidism (CH) is the most commonly seen endocrinological disorder of childhood. An increase in the prevalence of CH has been reported worldwide in the last 20 years.

**What this study adds?**

The rate of elevated neonatal thyroid stimulating hormone (TSH) was found to be 55% and approximately one of every two cases who were referred from national screening program was diagnosed with elevated neonatal TSH. Diagnosis was made in the first month in 87% of all cases. Dysgenesis and dyshormonogenesis rates were equal at 33.3%.

## Introduction

Congenital hypothyroidism (CH) is the most commonly encountered endocrinological disorder of childhood worldwide. Its incidence was reported as approximately 1/4000 in the 1970s when the screening program was first used. In the last 20 years, an increase in the prevalence of CH has been reported worldwide, possibly as a result of a gradual reduction in referral screening cut-off values (1).

In a study conducted in Turkey in 2003, the incidence of permanent CH was reported as 1/2512 (2). In an evaluation of data between the years 2008 and 2010, the incidence of CH was reported as 1/888 in 2008, 1/592 in 2009, and 1/650 in 2010 (3).

The absence of disease-specific clinical manifestation in CH at birth and the fact that complications can be prevented with early initiation of treatment within the first few weeks after delivery, plus the fact that treatment is effective and inexpensive, led to the establishment of national screening programs. A CH screening program was first implemented in 1974 in Quebec, Canada, and in Pittsburgh and Pennsylvania in the US (4,5). In Turkey, although local screening was conducted before, nationwide CH screening in conjunction with phenylketonuria screening was introduced for the first time on 25 December 2006 in Turkey (6,7).

In Turkey, a thyroid stimulating hormone (TSH) bloodspot threshold value of 20 mIU/L was used in the first years of screening and subsequently this value was decreased to 15 mIU/L in January 2009, upon detection of cases of missed diagnosis. However, in 2016, the screening cut-off TSH threshold value was again increased to 20 mIU/L (3,8). According to the guidelines of the Turkish CH national screening program, an infant is considered to have passed the screening test when the blood spot TSH concentration is <5.5 mIU/L. Values between 5.5 mU/L and 20 mIU/L, are reported for a second evaluation. All cases with a capillary TSH concentration above 5.5 mIU/L in repeat blood samples or above 20 mIU/L in the first sample are referred to the appropriate center for evaluation by venous thyroid function testing, including thyroxine (T4) and TSH.

In this present study, we aimed to investigate the rate of diagnosis and the time of diagnosis in cases with elevated TSH referred from the screening program; to evaluate the clinical and laboratory findings in these cases; and also to determine the etiological distribution among these patients.

## Materials and Methods

All infants referred to the Outpatient Clinic of Dr. Sami Ulus Obstetrics and Gynecology, Child Health and Disease Department of Ankara University, between the dates of September 30^th^ 2015 and April 1^st^, 2018, because of suspected CH following newborn screening were prospectively evaluated. The study was approved by the Local Ethics Committee (no: 45/2015). Prior to inclusion in the study, informed consent was obtained from all parents.

In our study, serum T_4_ and TSH levels were measured on the day of referral. The diagnosis and treatment plan was based on the Lawson Wilkins Pediatric Endocrine Society Guideliness for Congenital Hypothyroidism (9). Date of birth, date of admission, postnatal age, gender, gestational age in weeks, type of delivery and birth weight were recorded in all cases included in the study. Number, dates and results of heel prick tests were recorded. All cases were investigated for family and maternal history of thyroid disease, including a family history of CH. Consanguinity between parents, presence of iodine exposure in the mother and the baby and the iodine content of the salt used during pregnancy were recorded.

Body weight, height and head circumference were measured on admission. All patients were examined for clinical signs and symptoms associated with CH which included: lethargy, inactivity, hypotonia, difficulty in feeding, excessive sleepiness, constipation, abdominal distention, umbilical hernia, prolonged jaundice, galactorrhea, weak cry, hypothermia, cutis marmoratus, nasal congestion and dry and coarse skin. Physical examination was made for both additional congenital abnormalities and goiter. Serum thyroglobulin, TSH receptor binding antibody (TRB-Ab) and spot urinary iodine levels were measured in patients diagnosed with CH. The localization, volume and parenchymal echogenicity of the thyroid gland were evaluated by thyroid ultrasonography (USG). Thyroid gland localization and activity were evaluated by thyroid scintigraphy.

Serum TSH, free T_4_ (fT_4_), and free tri-iodothyronine (fT_3_) concentrations were measured by chemiluminometric method using an Advia Centaur XP (Siemens Healthcare limited-Oakville) device. Thyroglobulin concentration was measured by immunoassay method on the Siemens Immulite 2000 analyzer device with the immulite 2000-thyroglobulin kit (Siemens Healthineers-United States). TRB-Ab level was measured on the Berthold 1B2111 device (Berthold, USA) by radioimmunassay with a Beckmann Coulter RRA Anti-R TSH kit (Beckman Coulter Company-Czech Republic). Spot urinary iodine level was measured on Agilent 7500 ICP-MS device (Agilent Technologies, USA) by using ICP-MS analysis technique.

Thyroid USG was performed by a radiologist using a 7.5-MHz linear probe with a Toshiba Aplio 500 US device (Toshiba Medical Systems Co. Ltd, Otawara, Japan). Thyroid volume was calculated for each lobe using the following formula: (D1xD2xD3/1000) x 0.523 where D1 is the longest longitudinal, D2 is the anteroposterior and D3 is the largest transverse diameters, in cm, for each lobe and the total volume was calculated as the sum of two lobes in mL. Thyroid volumes for the neonatal period with a value of less than 0.64 mL (10^th^ percentile) in patients were considered to constitute hypoplasia, those with a volume greater than the 95th percentile value of 1.15 mL were considered to constitute hyperplasia and total volumes between 0.64 mL and 1.15 mL were considered normal (10). Thyroid scintigraphy was performed in the Nuclear Medicine Department of our hospital using General Electric Millenium device (General Electric; Elgems, Tirat Carmel, Israel) and gamma camera with technetium 99 pertechnetate.

### Statistical Analysis

In the presentation of descriptive statistics; the data obtained by measurement were expressed as mean±standard deviation and (minimum-maximum) and categorical data as number (percentage). Cross-table analyzes, Pearson and Fisher’s Exact chi-square tests were used to compare the qualitative characteristics of the groups. Compatibility to normal distribution of the numerical measurements in the groups was examined by independent groups for those with normal distribution in numerical measurements and with Mann-Whitney U test for those without normal distribution. IBM Statistical Package for the Social Science (SPSS; IBM Inc., Chicago, IL., USA) version 22 was used for all statistical analyzes. A p value of <0.05 was considered statistically significant.

## Results

Of the 109 newborns referred from the neonatal screening program a total of 60 (55%) infants were diagnosed; 52 (47.7%) at initial evaluation and eight (7.3%) at follow up. Twenty nine (48.3%) of patients with elevated neonatal TSH were female and female to male ratio was 1:1.07. Gender, gestational age, term/preterm rate, type of delivery and birth weight were similar in patients with elevated neonatal TSH compared to babies referred but who were not diagnosed with elevated neonatal TSH (non-CH group) (Table 1). Parental consanguinity rate was significantly higher (p=0.004) at 31.7% (19/52) in patients with elevated neonatal TSH compared to 8.16% (4/49) in the non-elevated neonatal TSH group. The family history of thyroid disease, family history of CH, the rate of iodized salt consumption and iodine exposure were similar in patients with elevated neonatal TSH compared to the non-elevated neonatal TSH group (Table 2).

One hundred patients (91.74%) required a second heel prick test. The mean number of heel prick test was similar in patients with elevated neonatal TSH and the non-elevated neonatal TSH group. For all referrals the first heel prick time was 1.97±1.58 days, the second heel prick time was 8.5±4.62 days and the third heel prick time was 15.9±5.43 days. The mean age at diagnosis in the elevated neonatal TSH group was 30.2±24.8 days; 22.13±10.35 days in 52 (86.7%) patients diagnosed as a result of initial evaluation and 82.62±28.53 days in eight (13.3%) patients diagnosed at follow up. Of the patients diagnosed on initial evaluation, two (4%) patients were diagnosed within the first seven days, three (6%) patients between eight and 14 days, 40 (76%) patients between 15 and 28 days and seven (13%) patients later than 28 days. Thus 87% of patients with elevated neonatal TSH were diagnosed and treatment initiated in the first month. Duration between the second heel prick time and the diagnosis was 13.98±9.97 days.

Clinical findings which are associated with hypothyroidism were detected in 19 (36%) of 52 infants diagnosed as a result of initial evaluation. There was lethargy in two (10.5%) patients, feeding difficulty in three (16.78%), constipation in six (31.5%), constipation and umblical hernia in one (5.26%), prolonged jaundice in six (31.5%) and weak cry in one (5.26%) patient. None of the patients diagnosed at follow up exhibited signs or symptoms associated with CH. Goiter was detected on physical examination in two patients diagnosed as a result of initial evaluation. There were concomitant abnormalities in 11 (18.2%) of all CH cases: four atrial septal defect (ASD), one PS, one hydrocephaly and meningomyelocele, two developmental dysplasia of hip and three patients had Down syndrome). The babies with Down syndrome all had cardiac abnormalities commonly associated with this condition; ASD, patent ductus arteriosus and ventricular septal defect.

Body weight, height and head circumference were similar on admission in patient diagnosed with elevated neonatal TSH and non-elevated neonatal TSH referrals. The mean TSH level was 79.5 (2-150) µIU/mL, mean fT4 level was 0.76 (0.12-1.68) ng/dL and mean fT3 level was 3.36 (0.36-5.01) pg/mL in patients diagnosed with elevated neonatal TSH. The fT4 level was low in 33 (55%) patients (<0.9 ng/dL) and normal in 27 (45%) patients (>0.9 ng/dL) on admission (Table 3).

Urinary iodine levels were measured in 29 patients and thyroglobulin concentration in 35 patients diagnosed as a result of initial evaluation. Urinary iodine level was normal in nine (31%) patients and high in 20 (69%) patient; thyroglobulin level was normal in five (14.3%) patients and high in 30 (85.7%) patients. TRB-Ab level was measured in 36 patients and it was negative in 32 (88.8%) patients, borderline positive in two (5.6%) patients and positive in two (5.6%) patients. Only one of the two patients who were TRB-Ab positive had autoimmune thyroid disease in maternal history.

Thyroid USG was performed in 49 (81.7%) infants diagnosed with elevated neonatal TSH at initial evaluation. The mean thyroid gland volume was 1.06±0.95 (0-3.74) mL. In three (6.12%) cases, the thyroid gland was not visualized in the normal location. One of these patients found to have sublingual ectopic thyroid gland and one of these patients found to have agenesis on thyroid scintigraphy performed before replacement treatment. Thyroid scintigraphy was not performed in the third baby but the thyroglobulin value was normal (16 ng/mL) in this case, so it was thought that there might be an inactivating mutation at TSH receptor. There was hypoplasia in 14 (29.16%) patients, normal gland in 16 (33.3%) patients and hyperplasia in 16 (33.3%) patients. Ectopic thyroid gland rate was found to be 2.08% (1/48), agenesis rate was 2.08% (1/48), hypoplasia rate was 29.1% (14/48) and total of thyroid dysgenesis rate was found to be 33.3% (16/48). Sixteen patients (33.3%) with hyperplastic thyroid gland were diagnosed as dyshormonogenesis, 16 patients (33.3%) with normal thyroid gland were diagnosed as transient hypothyroidism and/or possible dyshormonogenesis. The time of diagnosis, consanguinity rate, TSH, fT4, thyroglobulin and spot urinary iodine levels were similar in these three groups (Table 4).

Levothroxine was started at a mean dose of 9.34 (2.10-15.0) µg/kg/d in patients diagnosed with elevated neonatal TSH as a result of initial evaluation. TSH level normalization time after the treatment was 11.02 (4-30) days which equates to a postnatal age of 33.83 (13-70) days. Normalization of fT4 in those patients with low fT4 at diagnosis was a little faster at 9.03 (3-30) days after treatment initiation and by the postanatal age of 31.4 (19-56) days.

## Discussion

In our study, only 60 (55%) of 109 cases referred from the national screening program were diagnosed with elevated neonatal TSH. In a study conducted in our country, it was reported that 114 (44.5%) of 256 cases referred from national screening program were diagnosed with CH (11). In our study, female to male ratio was calculated as 1/1.07 in patients with elevated neonatal TSH. While previous studies have shown that female preponderance in female to male ratio in patients with CH was 1.8/1, 1.4/1 (12), it has been pointed out that the dominance of gender in recent years has shifted to male direction as 1:1.14, 1/1.16 (8,13). Dysgenesis is more common in female sex, whereas dyshormonogenesis is much lower in girls (13). In our study, low rate of cases diagnosed with dysgenesis and milder cases due to lowering TSH screening cut-offs may be the cause of variability in gender distribution.

On admission to hospital, the mean age of the cases was found 27.47±14.2 (7-70) days in our study and 24.54±13.46 (4-168) days in the study of Kor and Kor (8). The mean age at diagnosis was 30.2±24.8 days in all CH cases; 22.13±10.35 in patients diagnosed as a result of initial evaluation and 82.62±28.53 days in patients diagnosed at follow up in our study. Kor and Kor (8) reported in their study that 223 (96%) of all 233 patients were diagnosed as a result of initial evaluation and the mean age of diagnosis was 19.87±7.63 (4-51); 10 (4%) of patients were diagnosed at follow up and the mean age of diagnosis was 43.71±14.02 (29-65) days. The mean age of diagnosis was found as 19.7±8.30 (5-60) days in the study of Peltek Kendirci et al (11), 38.1±58 (4-342) days in the study of Kuşdal et al (14) and 23±14 days in the study of Simsek et al (15). Early diagnosis is the most important aim of the screening program. In Turkey, in a study comparing the pre-screening and post-screening diagnosis time, prior to the introduction of screening diagnosis occurred at a mean of 292 days whereas it decreased to 35.2 days after screening was introduced (16). Bongers-Schokking et al (17) reported that there was no neurodevelopmental difference in the patients with CH whose treatment was initiated with appropriate dose in the first 13 days compared to a healthy group. In our country, despite the screening program having been implemented for 12 years, diagnosis is still occuring later than in other national programmes, although there is marked variability from center to center. This is still a major improvement over the diagnosis times reported prior to the introduction of screening in Turkey. However, there is the delay between second heel prick test and first clinic visit is nearly 19 days. Further analysis of the causes of delay between referral and initial diagnostics visits would enable some remedial organisation to be put in place.

The mean TSH level was 54.8 (7.81-150) µIU/mL in patients diagnosed as a result of initial evaluation and 11.23 (2.02-12.9) µIU/mL in patients diagnosed at follow up in our study. The mean TSH level was found as 55.2±33.85 µIU/mL in the study of Peltek Kendirci et al (11), 15.8±28.69 µIU/mL in the study of Kor and Kor (8). Variability in individual TSH levels may be the cause.

In our study, the rate of presence of symptoms was 36.5% in patients with elevated neonatal TSH and the most common symptoms were constipation and prolonged jaundice. The rate of presence of concomitant congenital abnormality in patients with elevated neonatal TSH was found to be 18.3% (11/60). The most common abnormalities were cardiac malformations detected in seven cases (11.7%). Three patients (5%) were diagnosed with Down syndrome (diagnosed with CH subsequently). In the study of Razavi et al (18), the rate of accompanying abnormality was found to be 19.1% (30/157) which is similar to the rate we report. Razavi et al (18) also reported 4.6% of abnormalities being cardiac malformations and the rate of Down syndrome was 7.6% (12/157).

In our study, 16 patients (33.3%) with elevated neonatal TSH were diagnosed as dysgenesis. Hyperplasia was detected in 16 (33.3%) patients who were assigned a diagnosis of dyshormonogenesis. The majority of cases with normal thyroid gland will be diagnosed with transient hypothyroidism but some of them may later be diagnosed with thyroid dyshormonogenesis, so that it was concluded that the exact ratio can only be given when the patients are old enough to undertake a trial off T4 replacement therapy. Kor and Kor (8) reported that the etiological distrubition of CH was evaluated by thyroid USG and thyroid dysgenesis rate was found to be 28.6% of patients (24.5% hypoplasia, 4.1% agenesis), 71% of patients were found to be normal thyroid gland and 0.4% of patients were found to have hyperplasia. In the study of Kuşdal et al (14), 28.2% of patients had thyroid dysgenesis (10.2% agenesis, 10.2% hypoplasia, 5.2% ectopia, 2.6% hemiagenesis) and all the remaining cases had normal thyroid gland by USG evaluation (14). Özgelen et al (13) reported that the rate of dysgenesis was found to be 83.7% of the patients with CH (51.7% hypoplasia, 21.7% agenesis, 10.3% ectopia) and rate of dyshormonogenesis was found to be 10.3% in patients; etiological distribution in this study was made only among the cases with permanent CH and both USG and scintigraphy were used for etiological evaluation. Similarly, in the study of Bezen et al (19), etiological distribution was evaluated among the cases with permanent CH; rate of dysgenesis was found to be 52.2% (34.8% hypoplasia, 17.4% ectopia) and rate of dyshormonogenesis was found to be 47.8% in patients. Although there are many studies which have reported that the rate of dysgenesis is high (20,21), there has been an increase in the rate of dyshormonegenesis in recent studies in accord with our findings (19,22). An Italian national study conducted by Olivieri et al (12) reported the rate of dysgenesis in CH cases was 82% between years 1987 and 1998 although it decreased significantly compared to the previous period with a rate of 58% between years 1999 and 2006 due to significant chnages in the screening protocols used in Italy and, the authors speculate, the increased survival of many more premature infants. In the second period of the study, it was stated that the rate of detection of normal and hyperplastic thyroid gland increased compared to the first period, which again may be due to the changes in the Italian screning system and the greater proportion of unwell premature infants. In addition, it was noted that the rate of dyshormonogenesis was significantly higher in patients who had parental consanguinity (23). The consanguinity rate in our study was higher in patients with elevated neonatal TSH but there was no difference in consanguinity rate according to the etiology of elevated neonatal TSH In the group with dysgenesis, while consanguinity rate was 41.2%, it was 50% in the non-dysgenesis group (25% of whom had normal thyroid gland and 25% with hyperplasia). However, we think that the exact effect of consangunity in the etiological distribution of elevated neonatal TSH may be resolved by the evaluation of patients with permanent CH at follow up.

### Study Limitations

Thyroglobulin and urine iodine levels were not evaluated at diagnosis in all patients with elevated neonatal TSH. Imaging was not performed in all patients with elevated neonatal TSH.

## Conclusion

The rate of elevated neonatal TSH in the first month was 87%. The mean time of initiation of treatment was 22 (7-53) days. Dysgenesis rate was 33.3% and dyshormonogenesis rate was the same at 33.3%. The majority of cases with normal thyroid gland will be diagnosed with transient hypothyroidism but some of them may be diagnosed with thyroid dyshormonogenesis.

## Figures and Tables

**Table 1:**
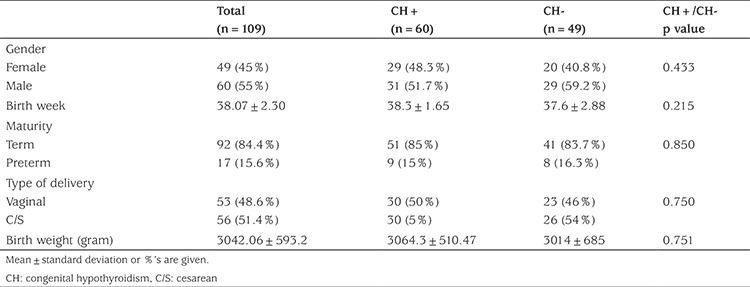
Clinical features of all referrals

**Table 2:**
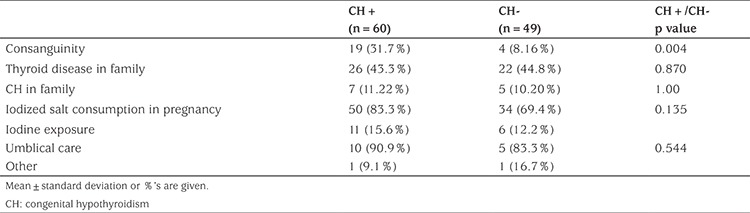
Demographic characteristics of congenital hypothyroidism (CH) group and non-CH group

**Table 3:**
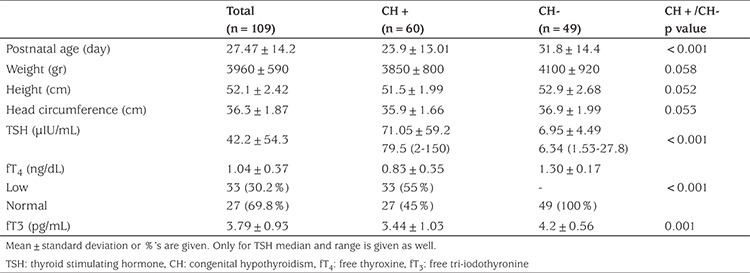
Postnatal age, anthropometric measurements and laboratory findings in all referrals at first assessment

**Table 4:**
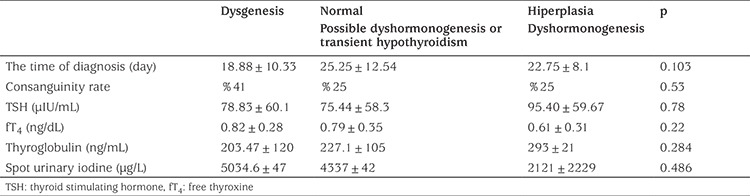
Postnatal age, anthropometric measurements and laboratory findings in all referrals at first assessment
